# On pollen and airborne virus transmission

**DOI:** 10.1063/5.0055845

**Published:** 2021-06-22

**Authors:** Talib Dbouk, Dimitris Drikakis

**Affiliations:** University of Nicosia, Nicosia CY-2417, Cyprus

## Abstract

This study investigates how airborne pollen pellets (or grains) can cause severe respiratory-related problems in humans. Given that pollen pellets can capture ribonucleic acid viruses, we show that airborne pollen grains could transport airborne virus particles such as the airborne coronavirus (CoV) disease (COVID-19) or others. We consider the environmental conditions featuring the highest pollen concentration season and conduct computational multiphysics, multiscale modeling and simulations. The investigation concerns a prototype problem comprising the transport of 10^4^ airborne pollen grains dropped from a mature willow tree at a wind speed of $\left(U_{\mathrm{wind}}=4\;\mathrm{km}/h\right)$. We show how pollen grains can increase the coronavirus (CoV) transmission rate in a group of people, including some infected persons. In the case of high pollen grains concentrations in the air or during pollination in the spring, the social distance of 2 m does not hold as a health safety measure for an outdoor crowd. Thus, the public authorities should revise the social distancing guidelines.

## I. INTRODUCTION

**FIG. 1.**
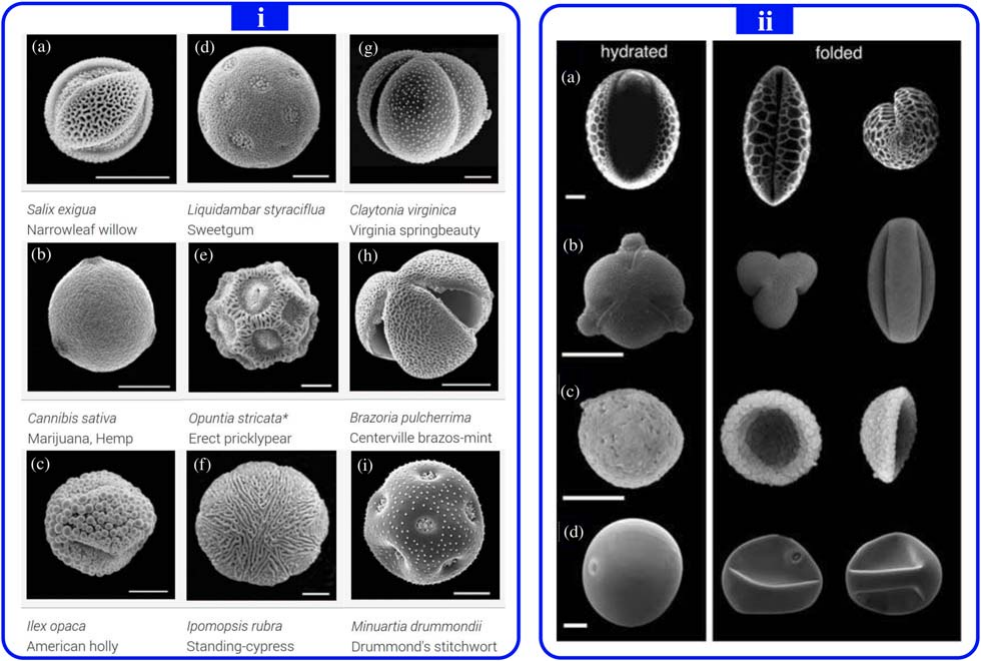
(i) An example of some pollen grains of different mythologies. All the white lines at the bottom of the images are equal to 10 *μ*m except the Opuntia, which is 25 *μ*. (ii) The effect of dehydration on folding pollen grains: (a) a monosulcate pollen grain of *Lilium longiflorum* in the hydrated state that witness aperture invagination in the folded state. (b) A tricolpate pollen grain of *Euphorbia milii* with the aperture that protrudes in the hydrated state but retracts completely within the pollen in the folded state. (c) Inaperturate pollen of *Aristolochia gigantea* with harmomegathy that is reduced to a mirror buckling of the pollen wall. (d) A monoporate pollen grain of maize (Zea mays). All the scale bars in (ii) correspond to 20 *μ*m. (i) Reproduced with permission from Arizona Board of Regents/ASU Ask A Biologist, https://askabiologist.asu.edu/images/zoom/pollen-gallery-pollen-close;^1^ (ii) Reproduced with permission from Katifori *et al.*, Proc. Natl. Acad. Sci. **107**, 7635–7639 (2017). Copyright 2017 Proceedings of the National Academy of Sciences.^2^

In plant biology, pollen is a microscopic grain discharged from the male part of a flower that can fertilize the female ovule to which pollen is transported in huge numbers by wind or in small numbers by insects such as bees. Pollination is the transport of pollen grains from their male site of production, i.e., tree and grass flowers, to the female landing site. Pollen grains size can range from some micrometers to tens or hundreds of micrometers. Their topology is very complex, and their shape generally varies from spherical to oval depending on the complex through a not fully understood dehydration process that occurs during pollination. Thus, the environmental conditions of the surrounding environment, i.e., ambient air temperature (*T_air_*), relative humidity (*RH*), and wind speed (*U_wind_*) play a crucial role in the pollination and the changing shape of pollen grains. Figure 1(i) shows an example of some pollen grains from electron microscopic images revealing different topologies.^1^ Figure 1(ii)^2^ shows an example of the shape change in pollen grains due to dehydration. Some of the airborne pollen grain species are known to cause human severe respiratory diseases.^3^

**FIG. 2.**
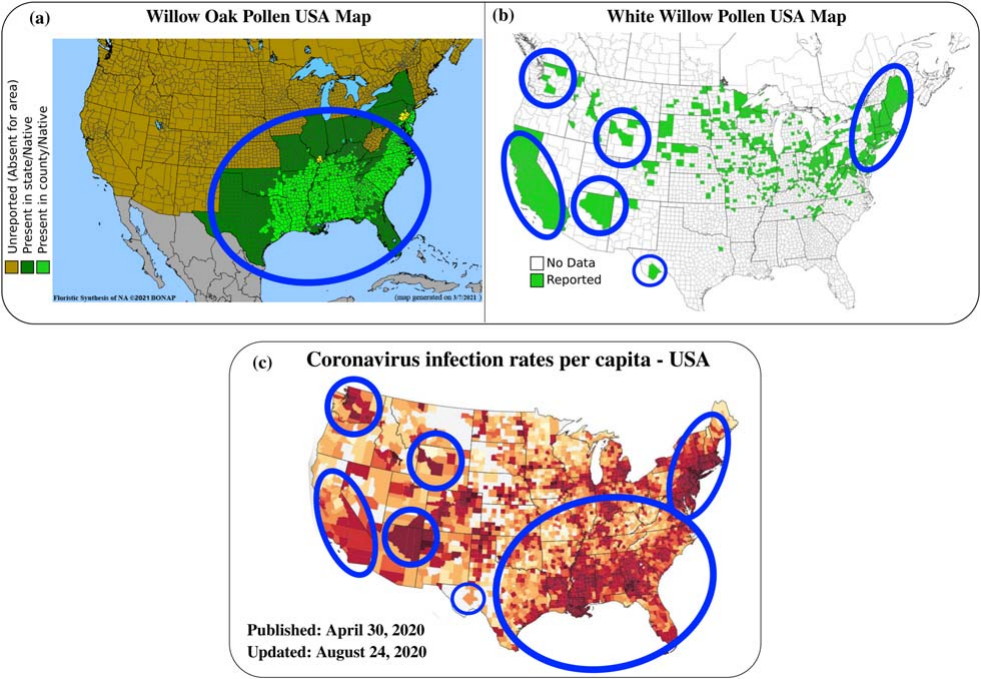
On the link between pollen maps (a) and (b) and the coronavirus infection rates per capita in the USA (c) published data on 30 April 2020.^15^ The links are highlighted by blue circles/ellipses. (a) The willow oak pollen map in the USA. Map used after permission from Ref. 16. The white willow (Salix Alba) pollen map in the USA.^17^ According to the pollen calendars by Lo *et al.* (2019),^14^ the highest concentrations of total allergic pollen grains in the USA and Canada (North America) are observed in the ambient air between March and May. (a) Reproduced with permission from the Biota of North America Program (BONAP) organization 2021, http://www.bonap.org/.^16^ (b) Reproduced with permission from the Early Detection and Distribution Mapping System (EDDMapS) 2021, https://www.eddmaps.org.^17^ (c) Reproduced with permission from the Big Local News COVID-19 map of 30 April 2020, https://covid19.biglocalnews.org/county-maps/index.html.^15^

The knowledge of airborne pollen transport, environmental effects, and the pollen interaction with other airborne viruses or aerosols is scarce. Madanes and Dadon^4^ and Velasco-Jiménez *et al.*^5^ made essential efforts to quantify the best sample location and the minimum required sample types of airborne pollen grains required to characterize site-scale airborne pollen. They found that increasing pollen counts from 150 to above 2700 does not yield different results among the dominant and subdominant types, which account for 70%–80% of the pollen sum. Sado and Inouye^6^ conducted a statistical study on airborne pollen grains. Plotting the number of days as a function of a specific type of airborne pollens, they found a normal distribution. Damialis *et al.*^7^ investigated the effects of wind direction, speed, and persistence on the transport of airborne pollen into a city. They revealed the significance of wind persistence in pollen transport, mainly when a breeze prevails for a considerable part of the year, i.e., the spring season. Tampieri *et al.*^8^ studied the transport of airborne pollen. Their work evaluated the area concerned with pollen dispersion coming from a distant source. They estimated, from travel time and the viability of the pollen itself, an extended distance within which the exchange of genetic material can occur. Buitink *et al.*^9^ investigated the calorimetric properties of dehydrating pollen by analyzing a desiccation-tolerant and an intolerant species.

The present study sheds light on the link between the increase in the coronavirus disease (COVID-19) infections among humans and pollination during March, April, and May, where specific outdoor temperature, relative humidity, and wind conditions exist. The above is based on the hypothesis that an airborne coronavirus (CoV) particle or airborne infected saliva droplet can adhere to a pollen grain. Past research supports evidence for the above as follows:

• Molecular detection of ribonucleic acid (RNA) viruses in pollen pellets.^10^
• Airborne pollen is likely a seasonal factor in inhibiting flu-like epidemics.^11,12^
• Airborne pollen grains can be present for a long period in the environment outdoor in cities but also indoor such as in street-level shops.^13^
• Data from pollen local distribution per capita in the United States of America (USA): for example, Fig. 2 shows the distribution of willow oak pollen and white willow pollen distribution, and coronavirus infection rates per capita in the USA. States of high pollen grains count witnessed higher coronavirus infection rates in the USA during the spring season (March–May 2020). According to the pollen calendars by Lo *et al.*,^14^ the highest concentrations of total allergic pollen grains in the USA and Canada (North America) are observed in the ambient air between March and May.

For the first time, through advanced computational multiphysics multiscale modeling and simulations, we investigate the effect of pollination on increasing airborne virus transmission. We will show how pollen grains dropped from a mature tree and transported by a breeze toward a crowd (including some infected individuals) can significantly contribute to an increase in the coronavirus transmission rate. Finally, we will emphasize the role of social distancing in reducing airborne virus transmission in the presence of airborne pollen.

## II. COMPUTATIONAL MODELS, DOMAIN AND BOUNDARY CONDITIONS

The Lagrangian multiphysics, multiscale computational fluid dynamics (CFD) solvers (transient numerical models) developed in Refs. 18–22 are applied in this study. We consider the coupled fluid dynamics and heat transfer equations at the scale of the pollen grains. Thus, we account for the effects of the change in calorimetric properties as a function of the relative humidity, temperature, and wind speed of the ambient air, e.g., particle size and drag and lift force variations due to the evaporation/dehydration of pollen grains.^9^ All the equations are discretized in space in a finite volume method^23^ framework, and the k−ω−SST turbulence model was applied as the turbulence model. The environmental conditions in the computational domain are imposed to values as those close to ones corresponding to a spring season with $T_{\mathrm{air}}=22\;{}^{\circ}C,2T_{\mathrm{ground}}=17\;{}^{\circ}C,1RH=50\%$. The boundary temperature of the crowd is applied as $T_{\mathrm{crowd}}=30\;{}^{\circ}C$.

**FIG. 3.**
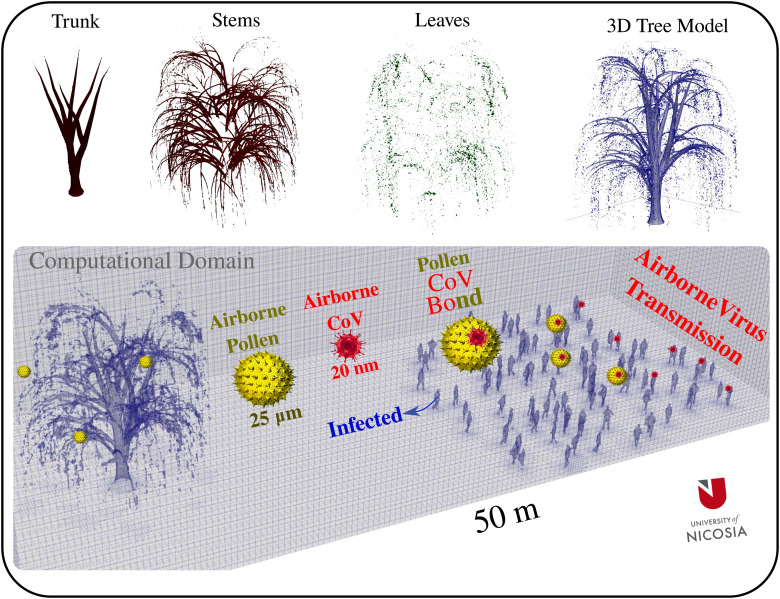
Top: computational modeling of a willow tree showing the trunk, stems, leaves, and the 3D tree model. Bottom: computational domain $\left(50\times 20\times 20\;m^{3}\right)$ that includes the tree model and a crowd of people (97 individual including some infected persons) illustrating the potential of airborne virus transmission through a pollen-CoV bond potential. The crowd's boundary is positioned at a distance of 20 m away from the tree's boundary. The clustering of people in the crowd respects, in general, the minimum social distance of 2 m.

A complete 3D model of a willow tree is created with a considerable number of tree leaves as sources of pollination (see Fig. 3) that ejects 10^4^ pollen grains due to a breeze at $U_{\mathrm{wind}}=4\;\mathrm{km}/h$. Furthermore, we introduce a realistic 3D model for a crowd of people into the computational domain at a vital distance away from the tree, as shown in the bottom side of Fig. 3. The velocity and pressure boundary conditions on the extremities of the computational domain are imposed as freestream boundary conditions. For pressure, this ensures an outlet–inlet condition that uses the velocity orientation to blend between zero gradients for standard inlet continuously and a fixed value for normal outlet flow: left side of the domain in Fig. 3 upstream of the tree. For the velocity, this ensures an inlet–outlet boundary condition that uses the velocity orientation to continuously blend between fixed value for standard inlet ( $U_{\mathrm{wind}}=4\;\mathrm{km}/h$) and zero gradients for outlet flow: right side of Fig. 3 downstream of the crowd.

We consider initially each pollen grain to have a density of ${\rho_{p}}_{0}=1435\;\mathrm{kg}\;m^{-3}$ and ${C_{p}}_{0}=2000\;J\;kg^{-1}\;K^{-1}$ corresponding to an initial 0.1 water content mass fraction (see Ref. 9). The initial size distribution of the pollen grains diameters attached to the tree leaves is imposed as a normal distribution law $f(d_{p})$ such that

$$f(d_{p})=\frac{1}{\sigma \sqrt{2\pi }}e^{\left(-\frac{1}{2}{\left[\frac{d_{p}-\mu }{\sigma }\right]}^{2}\right)},$$ (1)

with a mean value μ=25 μm and a variance σ=2 μm. These values are in the approximate correct statistical range of data for willow pollen grain diameters.

## III. RESULTS AND DISCUSSION

In this section, we present results for pollination for wind speed of $U_{\mathrm{wind}}=4\;\mathrm{km}/h\;(\approx 1.1\;m/s)$, air temperature $T_{\mathrm{air}}=22\;{}^{\circ}C$, ground temperature $T_{\mathrm{ground}}=17\;{}^{\circ}C$, and relative humidity RH=50%.

### A. Pollination: fluid flow dynamics

**FIG. 4.**
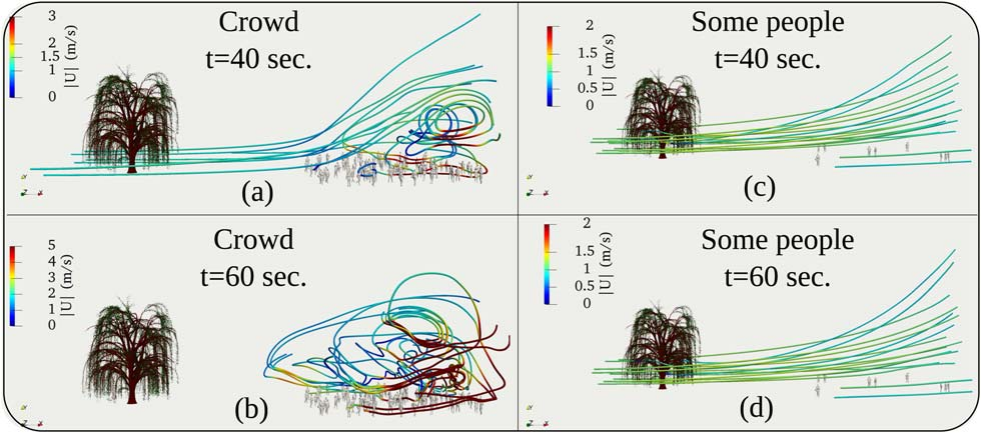
The three-dimensional streaklines (colored by the velocity magnitude) at t=40 s and t=60 s. (a) and (b) for a crowd of 97 individuals. (c) and (d) for a crowd of 11 individuals. The environmental conditions are free stream air flow at $U_{\mathrm{wind}}=4\;\mathrm{km}/h\;(\approx 1.1\;m/s)$. $T_{\mathrm{air}}=22\;{}^{\circ}C,\;T_{\mathrm{ground}}=17\;{}^{\circ}C,\;RH=50\%$.

**FIG. 5.**
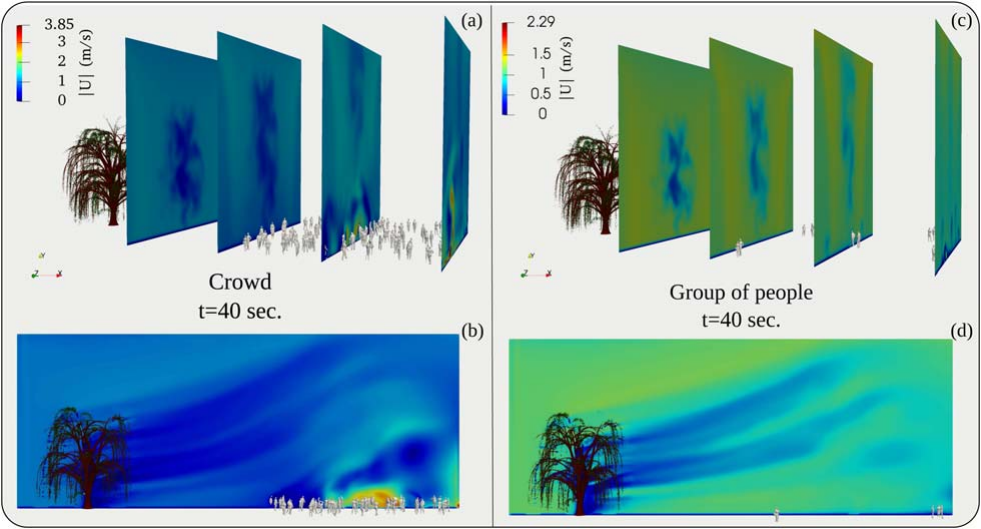
The velocity magnitude field at different cross sections in the computational domain. (a) and (b) for a crowd of 97 individuals. (c) and (d) for a crowd of 11 individuals. The environmental conditions are free stream air flow at $U_{\mathrm{wind}}=4\;\mathrm{km}/h\;(\approx 1.1\;m/s)$. $T_{\mathrm{air}}=22\;{}^{\circ}C,\;T_{\mathrm{ground}}=17\;{}^{\circ}C,\;RH=50\%$.

We performed simulations for two crowd scenarios comprising 11 and 97 people, located 20 m away from the tree at a wind speed of 4 km/h. These two crowd values represent, respectively, order of magnitudes of O(10) and O(100) that we bought from a commercial online vendor of real human model 3D surface creator.^24^ The numerical results show that the more significant number of people traps the flow and results in recirculation regions [Figs. 4(a) and 4(b)]. The flow circulation in the case of 11 people is negligible [Figs. 4(c) and 4(d)]. However, there is a significant flow diversion upwards. Figure 5 also reveals higher wind speeds surrounding the larger group. We attribute the above behavior to several reasons:

• The denser and larger crowd creates a rougher surface over which flowing air is perturbed, leading to recirculation regions and turbulence.
• The more significant number of people creates several small gaps that trap the flowing air leading to vortical structures.
• The vortical structures have a downward effect on the deflection in the case of a larger crowd. The above scenarios provide a qualitative comparison of the effects of larger vs smaller crowds on the environmental flow.
• Thermal gradients induce buoyancy effects. Individuals have higher boundary temperature, $T_{\mathrm{air}}=30\;{}^{\circ}C$, compared to the ambient air $T_{\mathrm{air}}=22\;{}^{\circ}C$.

### B. Pollination: Airborne virus transmission

**FIG. 6.**
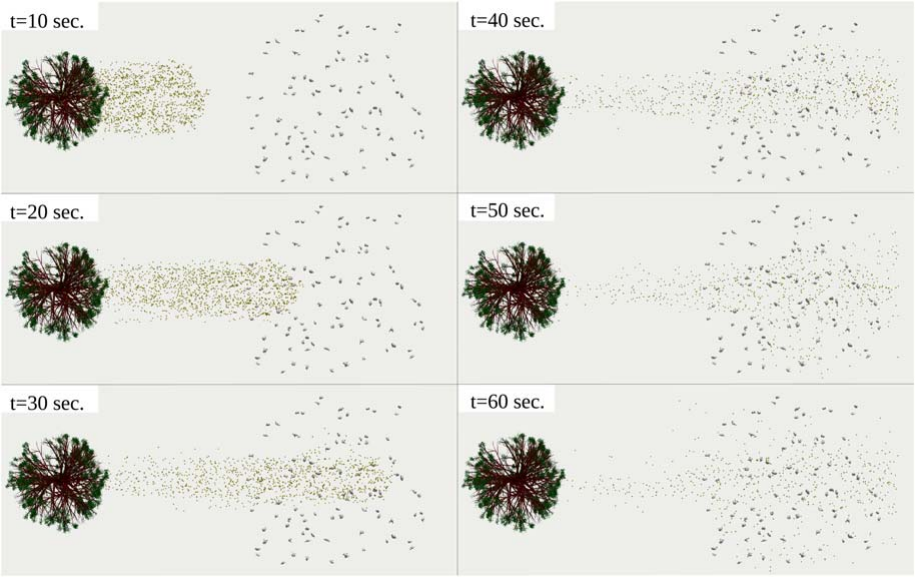
Top view of pollination of 10^4^ pollen grains detached from a willow tree at a breeze of $U_{\mathrm{wind}}=4\;\mathrm{km}/h$. The airborne pollen grains penetrate a crowd of 97 individuals with clusters that respect a minimum social distance of 2 m. Computational results at $T_{\mathrm{air}}=22\;{}^{\circ}C,\;T_{\mathrm{ground}}=17\;{}^{\circ}C,\;RH=50\%$. The pollen grains were scaled up by a factor of 5000 compared to their actual size. Multimedia view: https://doi.org/10.1063/5.0055845.110.1063/5.0055845.1

**FIG. 7.**
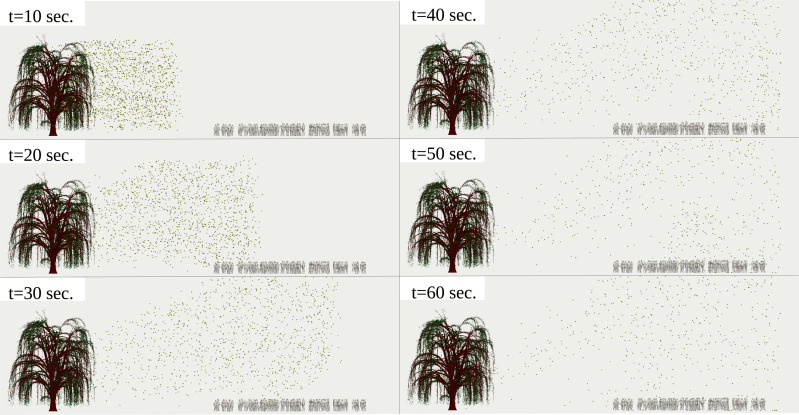
Side view of pollination of 10^4^ pollen grains detached from a willow tree at a breeze of $U_{\mathrm{wind}}=4\;\mathrm{km}/h$. The airborne pollen grains penetrate a crowd of 97 individuals with clusters that respect a minimum social distance of 2 m. Computational results at $T_{\mathrm{air}}=22\;{}^{\circ}C,\;T_{\mathrm{ground}}=17\;{}^{\circ}C,\;RH=50\%$. The pollen grains were scaled up by a factor of 5000 compared to their actual size. Multimedia view: https://doi.org/10.1063/5.0055845.1

**FIG. 8.**
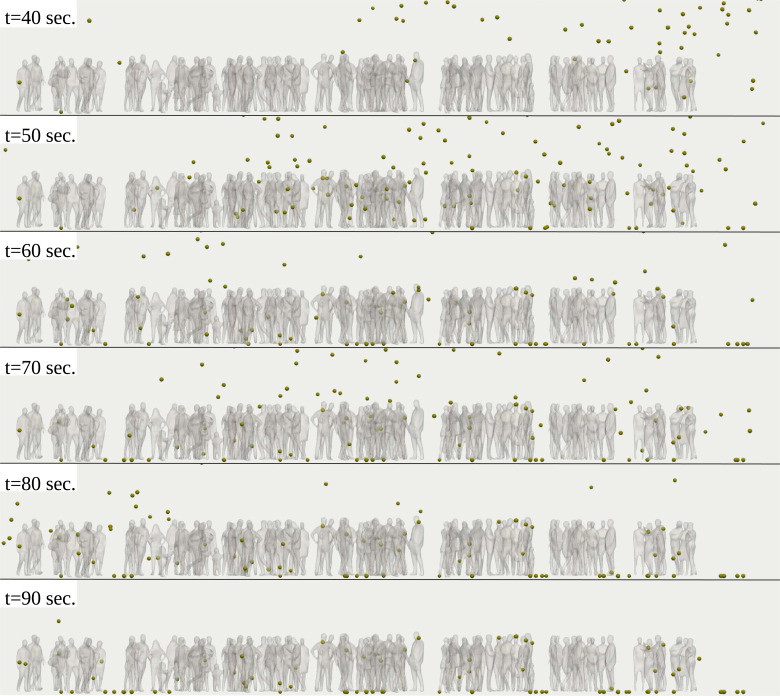
A zoom-in of the scene results of Fig. 7. Computational results at $T_{\mathrm{air}}=22\;{}^{\circ}C,\;T_{\mathrm{ground}}=17\;{}^{\circ}C,\;RH=50\%$. The pollen grains were scaled by a factor of 5000 compared to their actual size. Multimedia view: https://doi.org/10.1063/5.0055845.210.1063/5.0055845.2

The 10^4^ pollen grains released from the tree do not reach the crowd's boundary at t=10 s [Fig. 6 (Multimedia view)]. Some pollen grains start coming to the crowd's edge at t=20 s, consistent with the $U_{\mathrm{wind}}=4\;\mathrm{km}/h=1.1\;m/s$ speed of the breeze and the 20 m distance separating the crowd from the tree. At t=30 s, the pollen grains completely penetrate the crowd in the wind's direction (left to right) but without significant dispersion perpendicularly. A side perspective of the results is shown in Fig. 7 (Multimedia view). The pollen grains field continues to preserve its major flow direction, aligned with the breeze, without major dispersion in the gravitational direction. At t=40 s, t=50 s, and t=60 s, pollen grain dispersion takes place in all directions between the individuals [vertical and horizontal directions in Fig. 8 (Multimedia view)]. The above is due to thermal gradients between the individuals, the breeze, and the ground. If a virus infects some individuals, then a high concentration of dynamic airborne pollen grains in the air surrounding the individuals can increase the risk of airborne virus transmission. For example, when an infected person expels thousands of airborne infected saliva droplets, i.e., while talking, some of these contaminated droplets have a high probability of attaching to a surrounding pollen grain and thus being transported. We know that a pollen grain can be transported to longer distances with the wind than a saliva droplet. This is because a pollen particle is lighter, with less water content, and it is very much more porous than a saliva droplet. The above analysis leads to a critical conclusion that in environmental conditions like those in Figs. 6 and 7, pollen may increase the airborne virus transmission rate.

### C. Airborne pollen dynamics—Case of a group of people

**FIG. 9.**
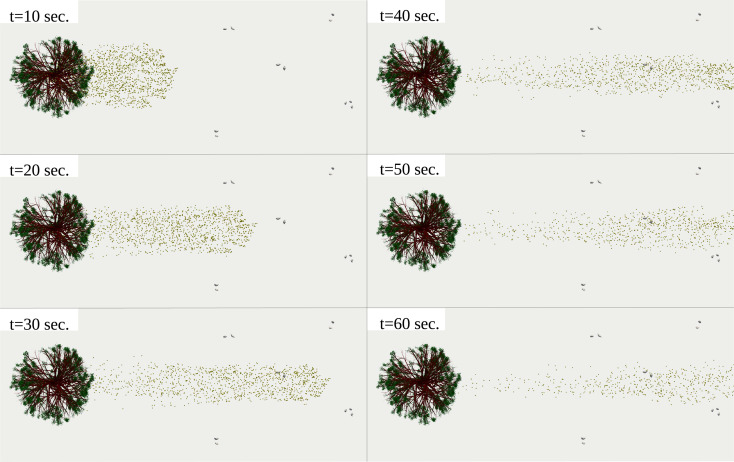
Side view of pollination of 10^4^ pollen grains detached from a willow tree at a breeze of $U_{\mathrm{wind}}=4\;\mathrm{km}/h$. The airborne pollen grains penetrate 11 individuals with clusters that respect a minimum social distance of 2 m. The environmental conditions are $T_{\mathrm{air}}=22\;{}^{\circ}C,\;T_{\mathrm{ground}}=17\;{}^{\circ}C$, and RH=50%. The pollen grains were scaled up by a factor of 5000 compared to their actual size. Multimedia view: https://doi.org/10.1063/5.0055845.310.1063/5.0055845.3

We investigate what happens if the crowd is reduced to only 11 individuals instead of 97 under the same breeze speed, temperature and boundary conditions. The results of Figs. 9 (Multimedia view) and 10 (Multimedia view) reveal that the pollen grains detached from the tree and transported by the wind do not disperse all around the areas, covering the individuals' positions. Therefore, in high pollen grain concentrations in the air or during pollination in the spring, the minimum social distance of 2 m does not hold as a safety measure for a crowd outdoor. Thus, it should be revised by public authorities.

**FIG. 10.**
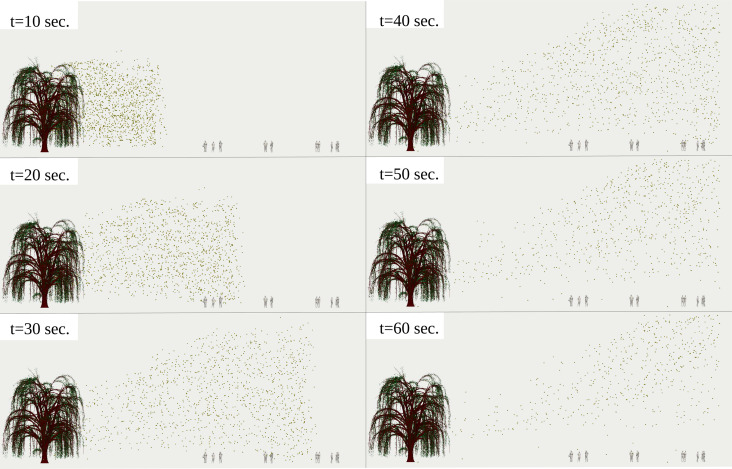
Side view of pollination of 10^4^ pollen grains detached from a willow tree at a breeze of $U_{\mathrm{wind}}=4\;\mathrm{km}/h$. The airborne pollen grains penetrate 11 individuals with clusters that respect a minimum social distance of 2 m. The environmental conditions are $T_{\mathrm{air}}=22\;{}^{\circ}C,\;T_{\mathrm{ground}}=17\;{}^{\circ}C$, and RH=50%. The pollen grains were scaled up by a factor of 5000 compared to their actual size. Multimedia view: https://doi.org/10.1063/5.0055845.410.1063/5.0055845.4

### D. Quantitative analysis

**FIG. 11.**
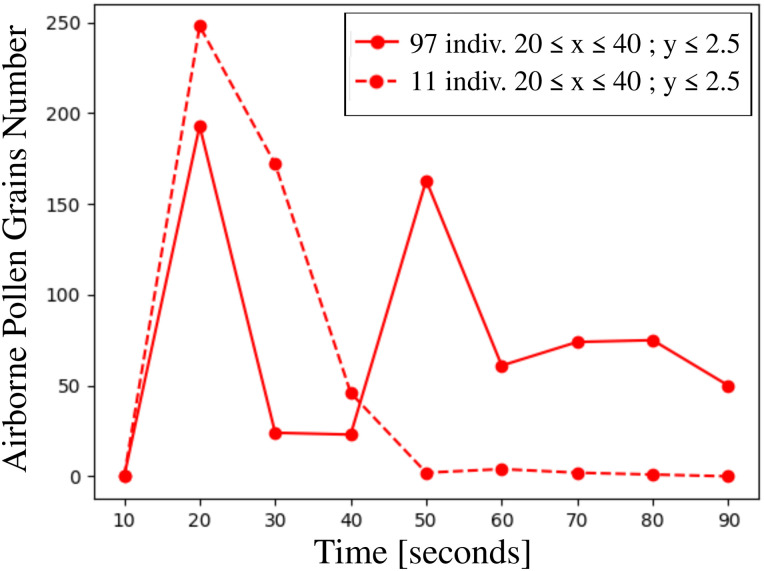
Total number of airborne pollen grains as a function of time inside the volumetric zone 20≤x≤40 m; y≤2.5 m that envelop the crowd of 97 individuals, and the group of people 11 individuals. The numerous pollen grains at the ground level *y *=* *0 are not counted. The simulations concern pollination of 10^4^ pollen grains detached from a mature willow tree at $U_{\mathrm{wind}}=4\;\mathrm{km}/h,\;T_{\mathrm{air}}=22\;{}^{\circ}C,\;T_{\mathrm{ground}}=17\;{}^{\circ}C,\;RH=50\%$.

**FIG. 12.**
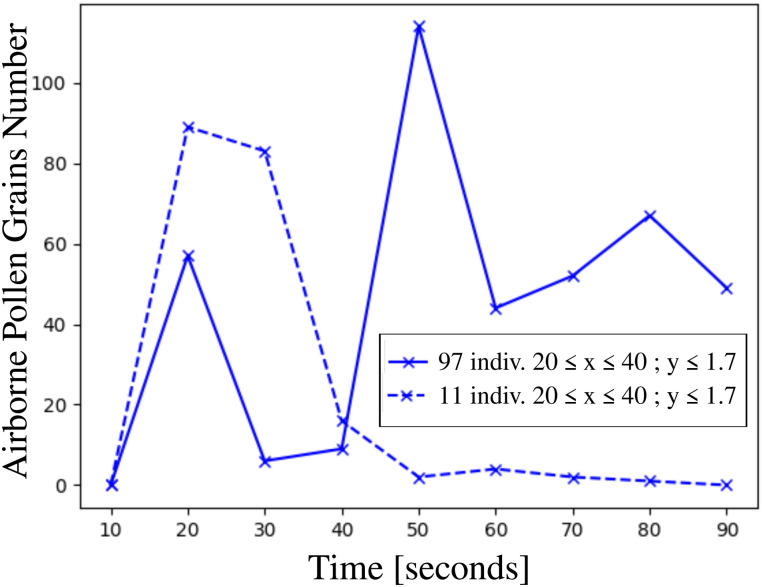
Total number of airborne pollen grains as a function of time inside the volumetric zone 20≤x≤40 m; y≤1.7 m that envelop the crowd of 97 individuals, and the group of people 11 individuals. The numerous pollen grains at the ground level *y *=* *0 are not counted. The simulations concern pollination of 10^4^ pollen grains detached from a mature willow tree at $U_{\mathrm{wind}}=4\;\mathrm{km}/h,\;T_{\mathrm{air}}=22\;{}^{\circ}C,\;T_{\mathrm{ground}}=17\;{}^{\circ}C,\;RH=50\%$.

**FIG. 13.**
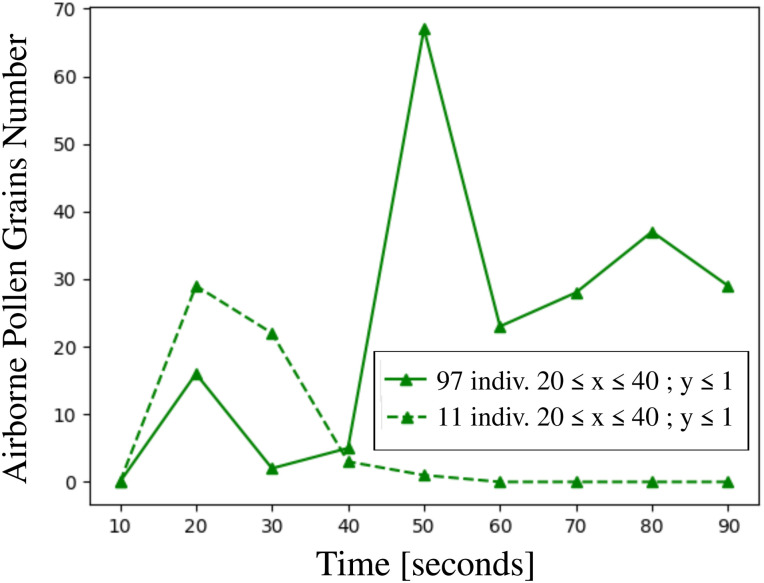
Total number of airborne pollen grains as a function of time inside the volumetric zone 20≤x≤40 m; y≤1 m that envelop the crowd of 97 individuals, and the group of people 11 individuals. The numerous pollen grains at the ground level *y *=* *0 are not counted. The simulations concern pollination of 10^4^ pollen grains detached from a mature willow tree at $U_{\mathrm{wind}}=4\;\mathrm{km}/h,\;T_{\mathrm{air}}=22\;{}^{\circ}C,\;T_{\mathrm{ground}}=17\;{}^{\circ}C,\;RH=50\%$.

We calculated the total number of airborne pollen grains suspended in the air in three different zones that envelop the crowd of 97 individuals and 11 individuals (Figs. 11–13). The numerous pollen grains at the ground level *y *=* *0 were not counted. In the volumetric zone at 20≤x≤40 m; y≤2.5 m, the pollen cloud traverses the group of people (Fig. 11) for ( t≥50 s). Both the residential time and the total number of pollen grains are higher in the crowd of 97 than 11 individuals. Thus, the risk of airborne virus transmission is higher in the case of a larger crowd.

Considering the zone 20≤x≤40 m and concentrate at lower heights (i.e., children, dogs presence) at y≤1.7 m and y≤1 m, Figs. 12 and 13 both reveal that when the pollen cloud traverses the group of people ( t≥50 s), the residential time and the total number of pollen grains are much higher in the case of the crowd. This finding indicates a higher risk of airborne virus transmission for children and animals present in the crowd.

## IV. CONCLUSION AND PERSPECTIVES

We showed that at some environmental conditions of ambient air temperature (*T_air_*), relative humidity (*RH*), and wind speed (*U_wind_*), airborne pollen grains play a non-negligible contributing role to transporting airborne virus particles. Under the scenario of a spring season conditions outdoor at $T_{\mathrm{air}}=22\;{}^{\circ}C,\;T_{\mathrm{ground}}=17\;{}^{\circ}C,\;RH=50\%$, we investigated the transport of 10^4^ airborne pollen grains dropped from a mature willow tree due to a breeze at $U_{\mathrm{wind}}=4\;\mathrm{km}/h$.

Assuming that a coronavirus particle can adhere to a pollen pellet's surface,^10^ we showed how airborne pollen pellets transported by a light wind can penetrate and disperse into a crowd of individuals, even when the social distance of 2 m is maintained. We showed that if only some persons in this crowd are infected, then the pollen grains dynamics can increase the airborne virus transmission rate.

Studying a smaller group of people under the same conditions, we found that the minimum social distance of 2 m is not sufficient as a health safety measure for a crowd outdoor at high pollen grain concentration or during pollination in the spring, i.e., March to May. Thus, public authorities should revise the social distancing recommendations. The above should be adapted accordingly in different countries, which may have different pollen seasonality.^25^

Future work will investigate the influence of different environmental conditions, i.e., parameters like the air temperature, relative humidity, and wind speed on the airborne virus transmission rate induced by pollination with critical pollen grain concentrations in the atmosphere.

## Data Availability

The data that support the findings of this study are available on request from the corresponding author upon reasonable request.
